# Bryophytes of the Loess Cliffs in the Pannonian Area of Austria

**DOI:** 10.3390/plants14203128

**Published:** 2025-10-10

**Authors:** Harald G. Zechmeister, Michaela Kropik

**Affiliations:** Department of Botany and Biodiversity Research, University of Vienna, 1030 Vienna, Austria; michaela.kropik@univie.ac.at

**Keywords:** *Hilpertia velenovskyi*, *Pterygoneurum compactum* complex, rare and endangered species, carbonate-related community structure, vegetation syntaxonomy

## Abstract

Austrian loess cliffs represent unique habitats supporting a rich bryophyte flora, including numerous rare and endangered species. We conducted a comprehensive survey of 86 loess cliff sites in the Pannonian area of Lower Austria, Burgenland, and Vienna, recording 79 bryophyte species. The results highlight that Austrian loess cliffs, despite their small spatial extent, are key refugia for light-demanding, desiccation-tolerant bryophytes. *Hilpertia velenovskyi*, a critically endangered species, was recorded at six new sites, expanding its known Austrian distribution. Our study also documents the first Austrian occurrences of several *Pterygoneurum* species. Seven bryophyte communities were distinguished: *Aloinetum rigidae*, *Hilpertio velenovskyi–Pterygoneuretum compacti*, and the newly described subassociations *Didymodontetum glauci didymodontetosum cordati* and *Eurhynchietum schleicheri didymodontetosum cordati*, as well as *Pterygoneuro–Acaulonetum triquetri* ass. nov. Multivariate analyses suggest carbonate content as the most consistent environmental driver. Despite their ecological significance, loess cliffs are increasingly threatened by habitat loss, overgrowth by vascular plants, and shading from invasive trees. Our study provides a detailed syntaxonomic and ecological framework for bryophyte communities on loess cliffs, underlining their role as refugia for rare species and the urgent need to protect remaining sites in the Pannonian region.

## 1. Introduction

Loess is an aeolian sediment [1], reshaped by processes of loessification [2,3]. It is characterized by a dominant coarse pore structure and a variable carbonate content, typically around 20–30% but occasionally as low as 5% [4,5,6]. Loess is primarily composed of silt, with a variable clay fraction (5–20%; [2]). Although generally bright in colour, it often appears yellow to reddish due to iron hydroxide. Nevertheless, loess exhibits considerable heterogeneity in carbonate content, mineral composition, grain size distribution, and sediment thickness, depending on regional setting, stratigraphic position, provenance, weathering intensity, and the development of loess–palaeosol sequences [6,7,8]. Loess deposits occur on every continent except Antarctica, covering nearly 10% of the Earth’s surface, particularly in the Northern Hemisphere [7].

In Austria, as in many other parts of the world, loess was predominantly deposited during cold phases of the Late Pleistocene, especially during the Würm glaciation [9,10,11]. However, palaeosols indicate that loess formation began as early as the Late Pliocene [12]. In the study area, most loess material derives from the alluvial plains of large rivers, especially the Danube. During cold periods, these areas were sparsely vegetated and thus highly susceptible to wind erosion, leading to the accumulation of loess up to 40 m thick. In Austria, loess covers approximately 1210 km^2^ [13], with the vast majority (90%; [14]) located in the east, particularly in the Weinviertel region of Lower Austria.

Loess represents the most important substratum in eastern Austria, forming the basis of agricultural landscapes. Because of its high water-holding capacity and nutrient availability, loess provides excellent soil for agriculture, particularly arable farming and viticulture [14]. Many vineyards in eastern Austria are situated on terraces with prominent loess walls. The surprisingly high stability of loess, despite being an unconsolidated sediment, allows the formation of vertical cliffs [9,12]. While loess cliffs naturally developed primarily along riverbanks, most of those present in Austria today are anthropogenic in origin, formed through hollow ways or terrace construction for viticulture [15,16]. Like viticulture itself, the vegetation of loess walls is closely tied to warm climatic conditions. Loess cliffs represent extremely dry habitats [15] and support xerophytic bryophyte communities adapted to semi-desert conditions. In rare cases, affinities to true semi-desert bryophytes can be observed [17], particularly in growth forms [18,19].

Loess deposits represent one of the most widespread Quaternary sediments worldwide, yet the bryophyte communities associated with them, particularly on vertical loess walls, remain poorly understood outside Europe. Only a few studies have addressed this topic beyond the continent, including investigations from China [20], the Sahara (e.g., [21]), and Iowa, USA [22]. In contrast, research in Pannonian Hungary has provided the most comprehensive insights, as loess walls occur there in high density along major river systems. These studies cover a wide range of ecological aspects: Pócs [15] described microtopographic and microclimatic conditions in detail, while Kürschner & Pócs [23] presented the most complete account of the bryophyte flora and its community associations. Additional studies examined life-form spectra [19,24] or focused on individual taxa [25,26]. A particular emphasis has been placed on *Hilpertia velenovskyi*, especially in Eastern European contexts [27,28,29], whereas other works reported more broadly on loess-associated bryophyte assemblages [30,31]. However, despite the broad distribution of loess landscapes across Central Europe, information from this region, especially from loess cliffs, remains very limited [32], and no systematic studies have yet been conducted in Austria.

Against this background, the present study addresses a major knowledge gap by providing the first systematic investigation of loess bryophytes in Austria. Specifically, we aimed to (i) characterize the bryophyte flora of Austrian loess cliffs in the Pannonian region, (ii) identify the ecological factors shaping their distribution, (iii) analyse species composition and provide a syntaxonomic classification, and (iv) place these findings within a broader international context. By combining floristic, ecological, and phytosociological perspectives, this study not only expands the biogeographic knowledge of loess-associated bryophytes but also provides a comparative framework for future conservation assessments at both regional and global scales.

## 2. Results

### 2.1. Bryophyte Species

**Figure 1:**
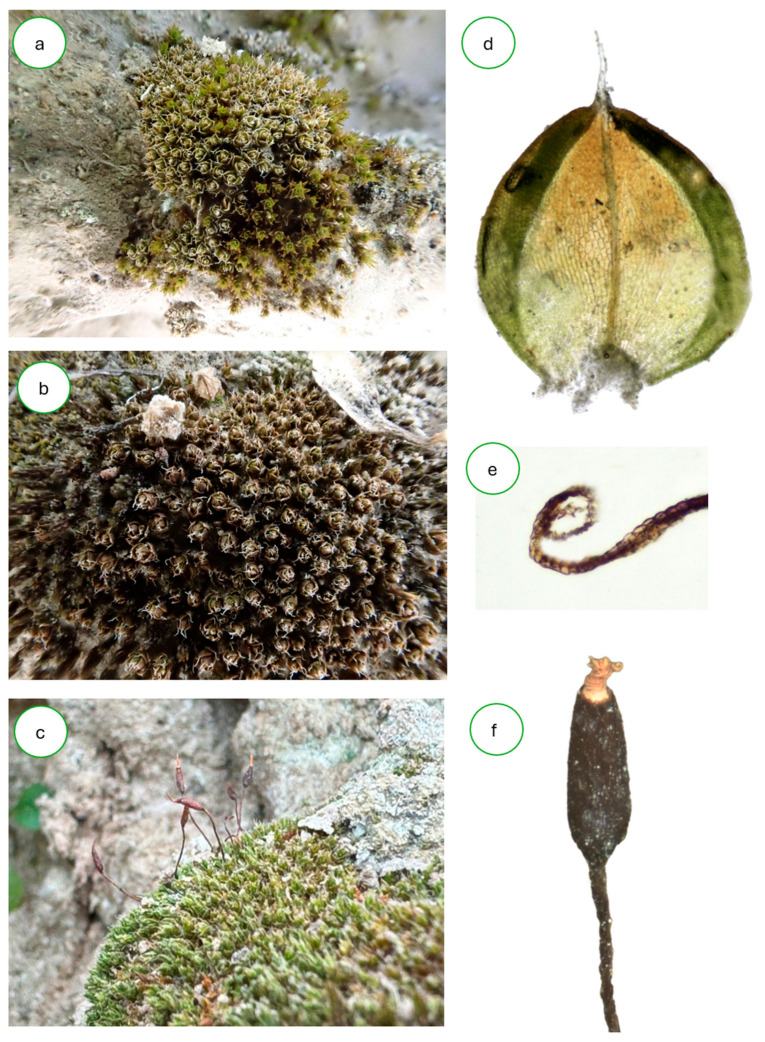
*Hilpertia velenovskyi*; (**a**–**c**) habitus from various sites; (**d**) leaf; (**e**) cross-section of a leaf showing the strongly involute leaf margin; (**f**) capsule with twisted peristome teeth.

A total of 79 bryophyte species were recorded (Table A1), all of them mosses except for *Fossombronia wondraczekii*. Among these, several species were first recorded for the Austrian flora, including *Microbryum rectum* and *Tortula brevissima*, which have already been published elsewhere [33]. In addition, the occurrences of *Pterygoneurum compactum* M.J.Cano, J.Guerra & Ros, *P. crossidioides* W.Frey, Herrnst. & Kürschner, and *P. squamosum* Segarra & Kürschner—if recognized as distinct species—constitute further first records for Austria, documented here for the first time. *Hilpertia velenovskyi* (Figure 1) is a particularly noteworthy element of the bryophyte flora in Austria and Europe. In addition to the previously known Austrian record near Kranberg [34], we report six new sites (Table 1).

According to the Red List of bryophytes for Lower Austria [35]—the most recent available, as no updated list exists for Austria as a whole—41% of the recorded species are classified as threatened. Of these, seven are Critically Endangered and nine Endangered (Table A1).

### 2.2. Site Characteristics

The loess walls surveyed had an average height of 4.2 ± 2.8 m (range: 1–15 m) and an average length of 38 ± 41 m (range: 2–300 m). Sites were situated at elevations between 140 and 338 m a.s.l. Mean annual temperature (2004–2024) was 11.2 ± 0.3 °C, with minimum winter temperatures averaging −12.1 ± 0.5 °C. The mean annual precipitation (2004–2024) was 580 ± 45 mm, occurring on 89 ± 6 days with >1 mm rainfall. Mean carbonate content was 24.4 ± 9.5%.

### 2.3. Syntaxonomic Classification

All relevés are provided in the Supplementary Materials (Tables S1–S4), with their syntaxonomic arrangement summarized in Table A2. Seven clusters of vegetation units were identified. One cluster (0; first TWINSPAN division) represented only a fragment and could not be assigned to any unit. The remaining clusters correspond to distinct vegetation units. Frequency is indicated as 1 = rare, 2 = scattered, and 3 = frequent (for details, see Methods).

***Eurhynchietum schleicheri* Waldh. 1944 *didymodontetosum cordati* subass. nov.** (Table S1, cluster 1).

Diagnostic species: *Oxyrrhynchium schleicheri*, *Oxyrrhynchium hians*, *Didymodon cordatus.*

Nomenclatural type (holotypus hoc loco): Lower Austria, district Hollabrunn, Großmeiseldorf, hollow way, NE exposition, 286 m a.s.l., 15.87574 East, 48.53305 North; Table S1, cluster 1, rel. Nr. 23, 1.0 m^2^.

Nomenclatural type relevé: *Oxyrrhynchium schleicheri* 2, *Oxyrrhynchium hians* 2, *Amblystegium serpens* 2, *Brachythecium rutabulum 2*, *Didymodon cordatus* 3, *Tortula lindbergii* 2, *Aloina ambigua* 2, *Streblotrichum convolutum* var. *convolutum* 2, *Bryum argenteum* 2, *Barbula unguiculata* 3, *Didymodon rigidulus* 2, *Ptychostomum imbricatulum* 2, *Tortula truncata* 2, *Ptychostomum capillare* 2, *Campyliadelphus chrysophyllus* 2.

The characteristic species of this community is *Oxyrrhynchium schleicheri*, which is relatively widespread in the lowlands of eastern Austria and occurs here with high constancy and fidelity. It can be distinguished from *O. hians* by the tree-like growth of its creeping shoots and the often twisted leaf apex of the latter. *O. hians*, together with other pleurocarpous species such as *Amblystegium serpens* and *Brachythecium rutabulum*, is a constant companion and clearly differentiates this community from all other loess wall assemblages. This pattern reflects its occurrence in (semi)-shaded hollow ways, where pleurocarpous species are more competitive than the low-growing acrocarpous taxa that dominate sunny walls. The mean carbonate concentration of sites with this community was 17.3% (± 7.8%). The consistently high presence of typical sunny-wall species (*Didymodon cordatus*, *Aloina ambigua*, and *Tortula lindbergii*) justifies the description of a new subassociation.

***Didymodontetum glauci* Ahrens ex Marst. 2015 *didymodontetosum cordati* subass nov.** (Table S1, cluster 2).

Diagnostic species: *Didymodon glaucus*, *Didymodon cordatus*, *Pseudocrossidium hornschuchianaum*.

Nomenclatural type (holotypus hoc loco): Lower Austria, district Tulln, Fels am Wagram Kellergasse Zwerigraben, SE exposition, 235 m a.s.l., 15.81909 East, 48.45192 North; Table S1, rel. Nr. 9, 0.5 m^2^.

Nomenclatural type relevé: *Didymodon glaucus* 2, *Didymodon cordatus* 3, *Aloina ambigua* 1, *Tortula lindbergii* 2, *Pseudocrossidium hornschuchianum* 1, *Streblotrichum convolutum* var. *convolutum* 2, *Bryum argenteum* 2, *Barbula unguiculata* 1, *Abietinella abietina* var. *abietina* 2, *Aloina rigida* 1, *Ptychostomum imbricatulum* 1.

The characteristic species of this community is *Didymodon glaucus*. *Didymodon glaucus* is known as a rare species with the main distributional range in Europe [36]; however, it expands with a few findings to the Far East (Baikal Lake and Irkutsk in Siberia) [37]. The community is comparatively species-poor, averaging 11.5 species per relevé, and is characterized by bryophyte mats on substrates with a high carbonate content (28.3 ± 7.6%). Unlike previously described occurrences, our populations are confined to semi-shaded, open loess walls and show marked differences in accompanying species. We therefore propose a new subassociation, defined primarily by the differential species *Didymodon cordatus*, and to a lesser degree by *Aloina ambigua* and *Tortula lindbergii*. An assignment to the *Fissidention gracillifolii* Neum. 1971 corr. Marst. 2001 nom. cons. propos. appears inappropriate. Instead, inclusion in the *Grimmaldion fragrantis* is more consistent, given the presence of numerous diagnostic species of that alliance, which also act as differential taxa for our subassociation.

***Didymodonto-Acaulonetum triquetri* ass. nov.** (Table S2).

Diagnostic species: *Acaulon triquetrum*, *Bryum dichotomum*, *Didymodon acutus*, *Tortula brevissima*, *Homalothecium lutescens.*

Nomenclatural type (holotypus hoc loco): Lower Austria, district Hollabrunn, Jetztelsdorf, Hausweingärten, anthropogenic loess cliff, SW exposition, 2 m high, 15 m long; 250 m a.s.l., 1,605,741 East, 4,872,576 North; Table S2, rel. Nr. 74, 1.0 m^2^.

Nomenclatural type relevé: *Didymodon cordatus* 3, *Acaulon triquetrum* 2, *Bryum dichotomum* 2, *Tortula brevissima* 1, *Homalothecium lutescens* 2, *Tortula lindbergii* 3, *Aloina ambigua* 2, *Streblotrichum convolutum* var. *convolutum* 3, *Pterygoneurum lamellatum* 2, *Pterygoneurum subsessile* 1, *Pterygoneurum ovatum* 3, *Barbula unguiculata* 3, *Ptychostomum imbricatulum* 2, *Bryum argenteum* 3, *Didymodon rigidulus* 2, *Didymodon fallax* 2, *Brachythecium rutabulum* 1.

This is the most species-rich community of the loess walls, with an average of 14.3 species per relevé. This community is the central element of loess communities. It is characterized less by specific species that only occur in this association than by the dominance of alliance species, a typical feature of ‘central’ communities. It is primarily developed on predominantly south-facing, anthropogenically created loess walls between vineyards, but also occurs on sunny, unshaded walls of hollow ways. The mean carbonate content of sites assigned to this community was 22.5% (±10.3%). Compared to the *Aloinetum rigidae* and the *Hilpertio velenovskyi–Pterygoneuretum compacti*, the loess of these stands retains higher moisture levels. The community frequently occupies the basal sections of the walls. The most constant species belong either to the *Grimmaldion* (e.g., *Didymodon cordatus*, *Tortula lindbergii*, *Aloina ambigua*, and *Streblotrichum convolutum*) or to the *Barbuletea unguiculatae* (e.g., *Pterygoneurum ovatum*). Within this community, *Acaulon triquetrum*, *Bryum dichotomum*, *Didymodon acutus*, and the rare *Tortula brevissima* show their main occurrence. *Homalothecium lutescens* provides a clear differential character against all other communities treated in this study. The community also forms a link to less steep loess surfaces, which, however, are not the focus of this study.

***Aloinetum rigidae* Stod. 1937** (Table S3, cluster 4).

Diagnostic species: *Aloina rigida*, *Didymodon cordatus*

This very species-poor community, with an average of 5.2 species per relevé, often occupies square-meter-sized patches, predominantly on very high loess walls of both natural and anthropogenic origin. The mean carbonate content of these sites was 25.3% (±8.9%). The characteristic species *Aloina rigida* shows a high constancy here, though always with relatively low cover, never exceeding 25%. It occurs only sporadically in other loess associations. Alongside this characteristic species, *Didymodon cordatus* is present in all relevés of this community with very high cover, dominating its overall physiognomy.

***Aloinetum rigidae aloinetosum obliquifoliae* subass. nov.** (Table S3, cluster 5).

Diagnostic species: *Aloina obliquifolia*, *Aloina rigida*, *Didymodon cordatus*, *Tortula lindbergii*

Nomenclatural type (holotypus hoc loco): Lower Austria, district Tulln, Großriedenthal, anthropogenic loess cliff, SE exposition, 2 m high, 30 m long; 264 m a.s.l., 15.88688 East, 48.47683 North; Table S3, cluster 5, rel. Nr. 14, 1.0 m^2^.

Nomenclatural type relevé: *Aloina obliquifolia* 2, *Aloina rigida* 1, *Didymodon cordatus* 3, *Tortula lindbergii* 2, *Pterygoneurum lamellatum* 1, *Pterygoneurum subsessile* 1, *Streblotrichum convolutum var. convolutum* 1, *Acaulon triquetrum* 1, *Aloina brevirostris* 1, *Pterygoneurum ovatum* 2, *Bryum argenteum* 2.

This subassociation differs from the typicum-association by the following features: (a) it is richer in species (9.2 species per relevé compared to 5.2), by the constant presence of *Aloina obliquifolia*, high constancy of *Tortula lindbergii* and *Pterygoneurum lamellatum*, and by its markedly lower carbonate content compared to the typical form (22.2 ± 9.9%).

***Hilpertio velenovskyi–Pterygoneuretum compacti* Kürschner & Pócs 2002** (Table S4).

Diagnostic species: *Hilpertia velenovskyi*, *Pterygoneurum compactum*.

As already described in detail by Kürschner and Pócs [23], this community is primarily characterized by the occurrence of the rare species *Hilpertia velenovskyi* and *Pterygoneurum compactum*. Although both species have only moderate constancy, they have a high degree of fidelity in this association. Besides some frequent *Grimmaldion* species, *Didymodon vinealis* has a focus on this syntaxon. Moreover, the loess walls where it occurs exhibit by far the highest carbonate content of all investigated sites (30.8 ± 5.7%), with a clear trend towards higher values than other communities (see below). The carbonate content of sites with *Hilpertia velenovskyi* was even higher (33.3 ± 3.2%).

**Figure 2:**
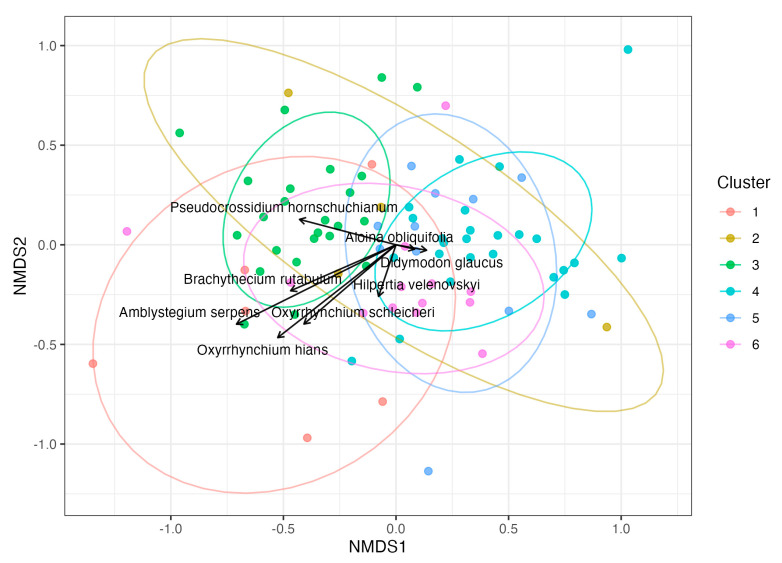
NMDS ordination (Bray–Curtis) of vegetation plots (Stress = 0.223). Arrows denote weighted-average positions of the top two diagnostic species per unit according to the group-standardized Phi coefficient (r.g; presence–absence; BH < 0.05; single groups only). Points are plots coloured by sociological units (1–6); ellipses show 68% normal-theory ellipses for groups with n ≥ 3.

The NMDS ordination (Bray–Curtis) showed modest but interpretable structure among plots (stress = 0.223). Indicator-species analysis (IndVal.g; single groups only; BH-adjusted) identified eight significant taxa (Table 2), including *Oxyrrhynchium schleicheri* and *Amblystegium serpens* (cluster 1), *Didymodon glaucus* (cluster 2) and *Pseudocrossidium hornschuchianum* (cluster 3), *Aloina obliquifolia* (cluster 5), and *Hilpertia velenovskyi* (cluster 6). These largely overlapped with the sociological reference list (five shared species), while four taxa were unique to the sociological set and three emerged exclusively from the statistical screening. A complementary Phi analysis (r.g on presence–absence; BH-adjusted) highlighted the top diagnostic species for each unit, which are displayed as weighted-average positions in the NMDS (Figure 2).

In contrast, the environmental signal was weak. Neither the full PERMANOVA on the standardized environmental matrix (Euclidean distance; *R*^2^ = 0.037, *F* = 0.586, *p* = 0.890; *N* = 82 complete cases) nor a core model restricted to mean annual temperature, annual precipitation sum, and carbonate content (*R*^2^ = 0.011, *F* = 0.167, *p* = 0.984; *N* = 84) indicated differences among units. Multivariate dispersion was also homogeneous (betadisper *p* = 0.491). At the univariate level, carbonate showed a nominal effect (Kruskal–Wallis *p* = 0.028), but this did not remain after BH correction across variables (*p*_BH = 0.227); all other variables were clearly non-significant.

## 3. Discussion

### 3.1. Discussion on Species and Communities

The syntaxonomic groups were created using largely traditional methods (e.g., [38,39]). The fact that we only used a three-level scale for frequency of occurrence did not influence the results. This approach and the resulting assignment to syntactic groups, as well as the resulting redefinition of syntaxa, are also fully consistent with the nomenclatural code [40]. Purely statistical methods, which do not require expert knowledge, have confirmed the majority of synsystematic classifications and the underlying characteristic species (Figure 2).

The IndVal analysis confirmed most sociologically defined character species and added several further significant indicators (Table 2). Notably, *Hilpertia velenovskyi*, a key species from a floristic–sociological perspective, also emerged as statistically significant, despite its patchy distribution across plots. This underlines its diagnostic value for loess cliff vegetation in Austria. A complementary Phi analysis (r.g) provided the top diagnostic species for each unit, highlighting those with the strongest fidelity even when their frequency across plots was limited. Together, both approaches produced a consistent picture: IndVal emphasizes constancy and overall representation across plots, while Phi identifies the most faithful markers of each unit. The overlap between both methods and the sociological reference list supports the robustness of our classification, while discrepancies highlight ecological variability and the nuanced behaviour of individual species.

The absence of significant environment–community relationships highlights that the floristic structure of loess-cliff bryophyte communities cannot be explained by simple abiotic gradients at the available resolution. Instead, their differentiation is better captured by floristic–sociological affinities, which reflect habitat microstructure, disturbance dynamics, and historical factors not encompassed by the measured variables. This finding underlines the strength of a combined sociological and statistical approach: sociological classification captures real floristic patterns even when major abiotic predictors are weak or absent.

***Didymodontetum glauci* Ahrens ex Marst. 2015 *didymodontetosum cordati* subass. nov.**

**Figure 3:**
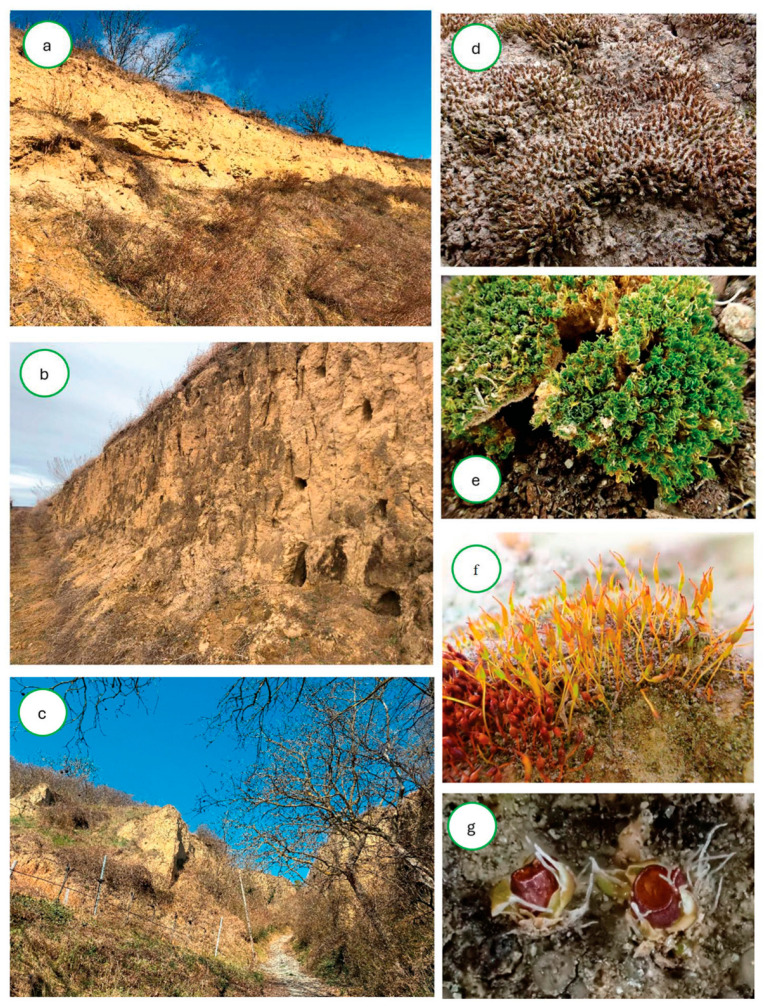
Selected sites and species from Pannonian loess cliffs in Austria: (**a**) Großebersdorf, (**b**) Untermarkersdorf, (**c**) Joching; (**d**) *Didymodon cordatus*, (**e**) *Didymodon glaucus*, (**f**) *Pterygonerum lamellatum* (center), *P. ovatum* s.str. (left corner), (**g**) *Pterygoneurum subsessile*.

*Didymodon glaucus* Ryan is a sub-Mediterranean–suboceanic species (Figure 3). Its habitats are usually described as shady and moderately moist [32,41,42]. These conditions do not correspond to our documented occurrences. Ahrens [43] characterizes the typical habitats of this community as very dry. This also applies to our relevés. Köckinger [44] reports occurrences in rain-sheltered, substrate-dry, often south-facing rock niches. This description matches the Austrian records more closely. The association has previously been assigned to the *Fissidention gracilifolii* Neum. 1971 corr. Marst. 2001, and thus to the *Ctenidietea mollusci* v. Hübschm. ex Grgic. [32]. However, this classification does not reflect the ecological conditions at our sites or the accompanying species of *D. glaucus*. We therefore assign this community to the *Grimmaldion fragrantis*.

***Pterygoneuro–Acaulonetum triquetri* ass. nov.**

This community is the central element of loess communities. Dierschke [45] describes communities that are characterized primarily by the occurrence of indicator species of a higher unit as ‘central communities’. This applies both to associations and their subunits, which are often then given the acronym ‘typicum’. This also applies to the newly described *Pterygoneuro–Acaulonetum triquetri*. This community also has the wide distribution in the European loess region required for central communitiesand it largely corresponds to what Marstaller [32] (p. 399) designates as the *Pterygoneurum ovatum*-community. In that unit, *Acaulon triquetrum*, *Bryum dichotomum*, and other *Grimmaldion* species occur with high constancy. Our relevés differ by the consistent presence of *Didymodon cordatus*, a typical indicator of vertical loess walls. This species is also dominant in Kürschner and Pócs [23], who refer to this unit as the “*Didymodon cordatus–Grimmaldion fragrantis* base community.” Their ecological descriptions correspond closely to ours. Ros and Guerra [46] describe similarities with the Mediterranean *Acaulon triquetri–Tortuletum brevissimae*. That community, however, includes Mediterranean species absent from our sites and is generally associated with drier conditions. By contrast, our relevés exhibit a slightly moister character and thus a higher species richness.

***Aloinetum rigidae* Stod. 1937.**

Drehwald and Preising [47] describe a subassociation with *Didymodon cordatus*. This reflects its dominance in our relevés. Later, Marstaller [48] treated it only as a variety. He argued that it is ecologically indistinguishable from the typical form. We follow this interpretation, although the conspicuous dominance of *D. cordatus* may be considered characteristic of loess walls. Most descriptions of this community are not based on loess cliffs. The exception is the work of Kürschner and Pócs [23].

***Aloinetum rigidae* Stod. 1937—*aloinetosum obliquifoliae* subass. nov.**

Marstaller (2023; [32]) describes *Aloina obliquifolia* as a “regional” character species; however, he notes that this could be a separate society. Due to the high consistency of loess cliffs indicator species, we follow this suggestion and elevate stands with *A. obliquifolia* to the rank of a subassociation.

***Hilpertio velenovskyi–Pterygoneuretum compacti* Kürschner & Pócs 2002.**

This association, first described by Kürschner & Pócs [23], is extremely rare in Austria. Its species composition and the carbonate content of its sites argue against inclusion in the *Aloinetum rigidae*, as suggested by Marstaller [32] for Müller’s [49] relevés. The association is clearly defined ecologically [23] and by constant companion species. Leaching and weathering increase carbonate content in the upper layers of loess walls [50]. These layers are also more exposed to the sun and wind, leading to greater desiccation. As a result, the community in Austria occurs predominantly at the top of loess walls. The distribution of *Hilpertio velenovskyi–Pterygoneuretum compacti* coincides with that of *H. velenovskyi* in Europe [23,31,51].

**Figure 4:**
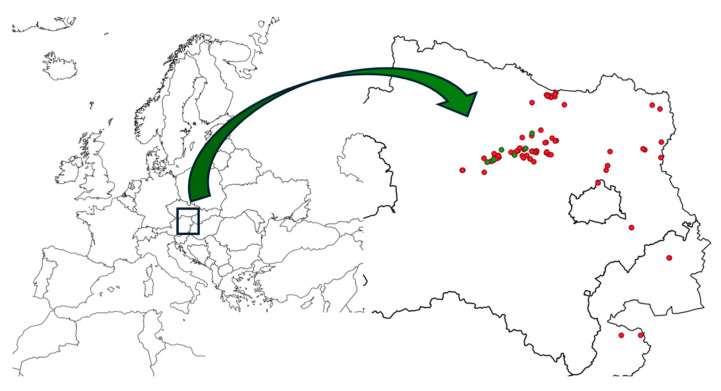
Map of sites of the investigated loess cliffs (red), dots in green are sites with *Hilpertia velenovskyi*.

*Hilpertia velenovskyi* (Schiffn.) R.H. Zander is the main characteristic species of very dry loess walls. It is a continental–subarctic element of cold loess steppes and occurs rarely and in scattered localities [28,29]. It is listed as Critically Endangered (CR) on the European Red List of bryophytes [52]. Before this study, only one Austrian record was known [34]. Our six new records considerably expand knowledge of its distribution in Austria. Specimens with sporophytes were observed at two sites, forming dense clusters ranging from 1 cm^2^ to 50 cm^2^ (Table 1). In Austria, the species is restricted to a narrow strip along the Wagram (Figure 4), following the Pleistocene Danube riverbed and its banks [53,54]. The same distribution pattern applies to the *Hilpertio velenovskyi–Pterygoneuretum compacti*, even at sites without *H. velenovskyi*. Austrian records connect seamlessly to the numerous Hungarian occurrences in the wider Danube region [29]. In Austria, however, the association occurs exclusively on anthropogenic loess cliffs.

Hodgetts et al. (2020; [55]) synonymize *Pterygoneurum ovatum* (Hedw.) Dixon with three additional species: *P. compactum* M.J.Cano, J.Guerra & Ros, *P. crossidioides* W.Frey, Herrnst. & Kürschner, and *P. squamosum* Segarra & Kürschner. In Central and South-Eastern Europe, however, these taxa can be distinguished morphologically and differ markedly in plant–sociological affinities. *Pterygoneurum compactum* is characteristic of the *Hilpertio velenovskyi–Pterygoneuretum compacti*. *Pterygoneurum squamosum*, recorded in three relevés, is also primarily associated with this community. In Hungary, however, it is characteristic of the subassociation *crossidietosum crassinervis* Kürschner & Pócs. In Austria, further differentiation is not possible. The diagnostic *Crossidium laxefilamentosum* has not been found, and the extremely dry and hot conditions of the Hungarian subassociation do not exist here. *Pterygoneurum crossidioides*, also rare, occurs consistently in all sun-exposed loess cliff communities. By contrast, *P. ovatum* s.str. is more unspecific. It is characteristic of the *Grimmaldion fragrantis* and represents a constant companion of all loess communities. Without differentiation of these four taxa, the sociological resolution of communities would become diffuse. We therefore recommend molecular analyses to clarify the taxonomic status of this species complex.

***Eurhynchietum schleicheri* Waldh 1944 *didymodontetosum cordati* subass. nov.**

Marstaller [32] reports this association among other locations from moderately acidic loess. His relevés, however, lack typical loess species. The carbonate content at our sites is the lowest among all loess communities. Marstaller describes the association as characteristic of shaded, warm-temperate deciduous forests and assigns it to the *Fissidention taxifolii* Marst. 2006, without further order- or class-level affiliation. Assigning this community to a higher synsystematic unit, therefore, remains problematic. *Fissidens* species and other companions noted by Marstaller are absent in our relevés. The high constancy of thermophilous loess species has led us to propose a new subassociation. Based on these species, an assignment to the *Grimmaldion fragrantis* Sm. et. Had. 1944 would also be conceivable. For now, however, we leave this decision open until further data are available.

A synoptic table of syntaxa observed at the Pannonian loess cliffs of Austria is given in Table A2. We suggest the following syntaxonomic scheme:

*Psoretea decipientis* Matt. Ex Follm. 1974
  *Barbulatalia unguiculatae* v. Hübschmann 1960
    *Grimmaldion fragrantis* Šm. et. Had. 1944
      *Didymodontetum glauci* Ahrens ex Marst. 2015 *didymodontetosum cordati* subass. nov.
      *Pterygoneuro-Acaulonetum triquetri* ass. nov.
      *Aloinetum rigidae* Stod. 1937
      *Aloinetum rigidae* Stod. 1937 *aloinetosum obliquifoliae* subass. nov.
      *Hilpertio velenovskyi-Pterygoneuretum compacti* Kürschner & Pócs 2002
    *Eurhynchietum schleicheri* Waldh. 1944 *didymodontetosum cordati* subass. nov.

### 3.2. Conservation Context

The remarkably high proportion of endangered bryophytes emphasizes the outstanding role of loess cliffs for bryophyte conservation (Table A1). The Red List of Lower Austria provides the most appropriate reference framework due to comparable biogeographic and climatic conditions. Whereas 29% of the regional bryophyte flora are classified as endangered, the proportion on loess walls reaches 41%. This striking difference highlights the exceptional conservation value of these habitats and is consistent with observations from other specialized substrates, where habitat restriction often correlates with higher extinction risk. Notably, several species are confined exclusively to loess walls, underscoring both their ecological specificity and vulnerability. At the same time, the extent of suitable cliffs has markedly decreased: in Lower Austria, approximately 70% of all hollow ways were destroyed or severely degraded between 1950 and 1990 [16]. These trends underline the urgency of integrating loess walls more explicitly into conservation planning, as their continued decline would disproportionately affect bryophyte diversity at both regional and supra-regional scales. Vertical loess cliffs adjacent to arable fields were flattened, which facilitated colonization by phanerogams, mainly grasses. These species displaced the light-demanding, desiccation-tolerant bryophytes. The growing expansion of photovoltaic installations in the immediate vicinity of, or even directly on, loess walls poses a significant threat not only to the rare loess-associated communities and their characteristic fauna (e.g., birds like the bee-eater, *Merops apiaster*), but also to the surrounding dry grasslands and traditionally managed vineyards that frequently accompany these habitats. Stochastic events, such as the frequent landslides in vertical loess cliffs, can also cause the rapid local extinction of rare species. Another serious threat is overgrowth and shading, particularly by Black Locust (*Robinia pseudoacacia*), which spreads at the base of the cliffs.

The conservation message is reinforced by our statistical results: since community composition is not strongly structured by broad climatic or edaphic gradients, the persistence of bryophyte communities depends primarily on habitat maintenance—especially the preservation of vertical cliff faces and the prevention of overgrowth.

## 4. Materials and Methods

**Study area:** According to Fink [56], the loess area of eastern Austria can be divided into three zones: “wet loess” in the west, “dry loess” in the east, and a “transition zone” between them. The transition zone is characterized by a notably higher carbonate content (20–40%) compared to the wet and dry loess zones, where carbonate rarely exceeds 10%. Exceptions occur east of Vienna (within the dry zone), where carbonate content may again reach 30% [6]. Smaller dry loess deposits are also found in Burgenland [57] and Vienna [58,59,60,61]. In Upper Austria [62] and Styria [63], only loess derivatives occur, namely loess-loam (also referred to as brown loess or dust loam), formed under humid climatic conditions.

**Climate:** The climate in which Austrian loess walls occur is predominantly Continental-Pannonian, with mean annual temperatures around 11 °C. Annual precipitation ranges from 350 to 650 mm, distributed across fewer than 50 rainy days. This climate favours a distinctive flora, with main growth periods in early summer and autumn. Bryophytes in this region grow primarily in winter, when mean temperatures range between 5 and 10 °C [64].

Climatic data for both historical and newly recorded sites were extracted from the SPARTACUS gridded dataset of the Central Institute for Meteorology and Geodynamics (ZAMG), which provides a spatial resolution of 1 km^2^ and a daily temporal resolution from 2004 to 2024 [65,66]. From these data, we calculated mean annual temperature, minimum and maximum annual temperatures (°C), and mean annual precipitation. To capture precipitation evenness, we additionally calculated the number of days with ≥1 mm precipitation, as bryophytes respond more favourably to regular light rainfall than to isolated heavy rain events.

**Carbonate content** data for the various loess areas were taken from Rabeder et al. [6]. These values refer to regional averages, meaning that the actual carbonate content at specific collection points may be somewhat higher or lower. Nevertheless, as the data are based on a large number of samples per region, they provide a reliable approximation of the conditions at the investigated loess walls.

**Field surveys** were carried out between December 2023 and April 2025, corresponding to the wet winter months. Previous studies [33,67,68,69,70] have demonstrated that this period provides optimal conditions for bryological fieldwork in the Pannonian region.

The entire loess area of eastern Austria was surveyed, although in many parts, loess walls were absent due to geomorphological conditions and agricultural practices. Sites were randomly selected by exploring geomapped loess areas (via car and extended hikes) and by including locations reported in floristic and geological literature. In regions with a high density of walls, sites were chosen at random to represent the full range of wall heights, ensuring a representative coverage of loess regions in eastern Austria. In total, 86 sites with loess cliffs were analysed: 80 in Lower Austria, 4 in Burgenland, and 2 in Vienna. Taking into account the variation in loess types [6,56], six sites were situated in the wet zone, 27 in the transitional zone, and 53 in the dry zone, accurately reflecting the relative extent of these zones. Site locations are shown in Figure 4, with the map generated using QGIS version 3.3 [71].

Loess walls were surveyed in their entirety, with only accessible areas investigated in cases of extreme wall height. Relevés were recorded in homogeneous sections, occasionally distinguishing between wall surfaces and bases. Species abundance at each wall was assessed using a three-point scale (frequent ≈ >20% cover, scattered ≈ 2–20%, rare ≈ <2%), following the International Code of Phytosociological Nomenclature [40], which stipulates a minimum three-class scale for valid syntaxon description. More detailed quantification of cover values was deliberately avoided, as the small size of mosses and their frequent co-occurrence with congeners—often separable only through microscopic analysis—would render finer estimates misleading.

**Data processing**: Vegetation relevés were processed with TURBOVEG [72] and analysed for phytosociological correlations using JUICE [73]. For classification, non-classified TWINSPAN [38] was applied using three pseudospecies cut levels and four levels of division. Relevés showing a mismatch between the total cover of diagnostic species and the initial, TWINSPAN-based assignment were manually re-assigned. We used diagnostic species to establish a formal definition of the various syntaxa (e.g., [39]).

**Ordination of species composition**: We summarized community composition by non-metric multidimensional scaling (NMDS) based on Bray–Curtis dissimilarities in two dimensions. Ellipses represent 68% normal-theory contours and were drawn only for sociological classes with at least three plots to avoid unstable estimates.

**Environmental differentiation among sociological classes**: We tested whether sociological classes differed in environmental space using PERMANOVA (adonis2) on Euclidean distances of the standardized eight-variable environmental matrix (minimum annual temperature, maximum annual temperature, mean annual temperature, annual precipitation sum, carbonate content, elevation, wall height, and wall length). We used 9999 unrestricted permutations and report *R*^2^, *F*, and permutation *p*. To rule out dispersion artefacts, we tested homogeneity of multivariate dispersion with betadisper (permutation test of distances to class centroids). As a sensitivity analysis, we repeated PERMANOVA for a reduced set of key variables (mean annual temperature, annual precipitation sum, and carbonate content).

**Univariate environmental comparisons**: For each environmental variable, we tested for differences among sociological classes using Kruskal–Wallis tests, with Benjamini–Hochberg (BH) correction across variables.

**Indicator species analysis and diagnostic plotting**: Indicator species were identified with IndVal.g based on abundance data, restricting associations to single sociological units only (duleg = TRUE), with 9999 permutations. Species-wise *p*-values were BH-adjusted across all tested species; only BH-adjusted *p* < 0.05 were retained (Table 2). As a complementary measure of diagnosticity, we calculated the group-standardized Phi coefficient (r.g) on presence–absence data, again restricted to single units, with 9999 permutations and BH-adjusted *p*-values. For visualization, significant IndVal species (and, separately, the top two Phi-diagnostic species per unit) were projected into the NMDS as weighted averages; arrows indicate centres of occurrence and are descriptive rather than inferential.

**Software**: All analyses outside JUICE were conducted in R version 4.4.3 [74] using the packages vegan, indicspecies, permute, dplyr, tidyr, ggplot2, and ggrepel.

**Nomenclature of bryophytes** follows Hodgetts et al. [55] unless otherwise stated. Specimens are stored in the private herbarium of the first author.

## Figures and Tables

**Table 1:** Sites with occurrences of *Hilpertia velenovskyi*, number of patches, and occupied area (cm^2^). The site Kranberg O Hollenstein (*) was already published in Zechmeister et al. (2017 [34]); all other sites are new records.

Site	Nr. of Patches	Area Occupied	Sporophytes
Kranberg O Hollenstein *	1	1	n
Ottenthal N	8	10	y
Gebling SW	15	40	n
Unterrohrendorf-Schnabl	20	30	n
Gedersdorf W	25	50	n
Fels am Wagram N	4	10	y
Straß im Straßerthal, Geißberg	1	5	n

**Table 2:** Significant indicator species (IndVal.g, single groups only, BH-adjusted, *n* = 9999 permutations). The table shows species, their associated unit (sociological class), IndVal statistic, and BH-adjusted *p*-values.

Species	Cluster	IndVal	*p*_BH
*Aloina obliquifolia*	5	0.906	0.002
*Amblystegium serpens*	1	0.816	0.002
*Didymodon glaucus*	2	1	0.002
*Oxyrrhynchium schleicheri*	1	0.899	0.002
*Brachythecium rutabulum*	1	0.804	0.005
*Oxyrrhynchium hians* var. *hians*	1	0.77	0.005
*Hilpertia velenovskyi*	6	0.734	0.013
*Pseudocrossidium hornschuchianum*	3	0.671	0.013

## Data Availability

Available data can be found in the Supplementary Materials.

## Supplementary Materials

The following supporting information can be downloaded at: https://www.mdpi.com/article/10.3390/plants14203128/s1, Table S1: *Eurhynchietum schleicheri* Waldh. 1944 *didymodontetosum cordati* subass. nov. (cluster 1), *Didymodontetum glauci* Ahrens ex Marst. 2015 *didymodontetosum cordati* subass. nov. (cluster 2), fragment community (cluster 0); C%–constancy in % of a species in the associated community, relevé number in bold: nomenclatoral type.; Table S2: *Didymodonto-Acaulonetum triquetri* ass. nov, C%–constancy in % of a species in the community, relevé number in bold: nomenclatoral type.; Table S3: *Aloinetum rigidae* Stod. 1937 (cluster 4); *Aloinetum rigidae-Aloinetosum obliquifoliae* subass nov. (cluster 5); C%–constancy in % of a species in the associated community, relevé number in bold: nomenclatoral type.; Table S4: *Hilpertio velenovskyi–Pterygoneuretum compacti* Kürschner & Pócs 2002 (cluster 6); C%–constancy in % of a species in the associated community.

## Appendix A

**Table A1:** List of species found in the Pannonian loess cliffs in Austria; Red Data status derived from Zechmeister et al. [35], expanded with additions (marked with *) in Zechmeister et al. [34], Zechmeister & Kropik [33].

Species	RL status
*Abietinella abietina* (Hedw.) M.Fleisch. var. *abietina*	LC
*Acaulon muticum* (Hedw.) Müll.Hal.	EN
*Acaulon triquetrum* (Spruce) Müll.Hal.	VU
*Aloina aloides* (Koch ex Schultz) Kindb.	* CR
*Aloina ambigua* (Bruch & Schimp.) Limpr.	EN
*Aloina brevirostris* (Hook. & Grev.) Kindb.	RE
*Aloina obliquifolia* (Müll. Hal.) Broth.	* EN
*Aloina rigida* (Hedw.) Limpr.	VU
*Amblystegium serpens* (L. ex Hedw.) Schimp.	LC
*Barbula convoluta* Hedw.	LC
*Barbula unguiculata* Hedw.	LC
*Brachytheciastrum velutinum* (L. ex Hedw.) Ignatov & Huttunen	LC
*Brachythecium albicans* (Neck. ex Hedw.) Schimp.	LC
*Brachythecium campestre* (Müll.Hal.) Schimp.	NT
*Brachythecium glareosum* (Bruch ex Spruce) Schimp. var. *glareosum*	LC
*Brachythecium rutabulum* (L. ex Hedw.) Schimp.	LC
*Bryum argenteum* Hedw.	LC
*Bryum dichotomum*	LC
*Bryum gemmiferum* R.Wilczek & Demaret	VU-R
*Bryum klinggraeffii* Schimp.	NT
*Bryum radiculosum* Brid.	VU
*Bryum violaceum* Crundw. & Nyholm	NT
*Campyliadelphus chrysophyllus* (Brid.) R.S.Chopra	LC
*Ceratodon purpureus* (Hedw.) Brid.	LC
*Dicranella howei* Renauld & Cardot	VU
*Didymodon acutus* (Brid.) K.Saito	LC
*Didymodon cordatus* Jur.	NT
*Didymodon fallax* (Hedw.) R.H.Zander	LC
*Didymodon ferrugineus* (Schimp. ex Besch.) M.O.Hill	LC
*Didymodon glaucus* Ryan	NT
*Didymodon insulanus* (De Not.) M.O.Hill	NT
*Didymodon rigidulus* Hedw.	LC
*Didymodon vinealis* (Brid.) R.H.Zander	EN
*Ditrichum pusillum* (Hedw.) Hampe	NT
*Encalypta streptocarpa* Hedw.	LC
*Encalypta vulgaris* Hedw.	LC
*Entodon concinnus* (De Not.) Paris	LC
*Fossombronia wondraczekii* (Corda) Lindb.	NT
*Funaria hygrometrica* Hedw.	LC
*Grimmia montana* Bruch & Schimp.	VU
*Grimmia pulvinata* (Timm. ex Hedw.) Sm.	LC
*Grimmia tergestina* var. *tergestinoides* Culm.	LC
*Hilpertia velenovskyi* (Schiffn.) R.H.Zander	* CR
*Homalothecium lutescens* (Hedw.) H.Rob.	LC
*Microbryum curvicollum* (Ehrh. ex Hedw.) R.H.Zander	VU
*Microbryum floerkeanum* (F.Weber & D.Mohr) Schimp.	EN
*Microbryum rectum* (With.) R.H.Zander	* CR
*Microbryum starckeanum* (Hedw.) R.H.Zander	EN
*Orthotrichum anomalum* Hedw.	LC
*Oxyrrhynchium hians* (Hedw.) Loeske var. *hians*	LC
*Oxyrrhynchium schleicheri* (R.Hedw.) Röll	LC
*Plagiomnium cuspidatum* (Hedw.) T.J.Kop.	LC
*Plagiomnium undulatum* (Hedw.) T.J.Kop.	LC
*Pseudocrossidium hornschuchianum* (Schultz) R.H.Zander	LC
*Pseudocrossidium revolutum* (Brid.) R.H.Zander	EN
*Pterygoneurum compactum* M.J.Cano, J.Guerra & Ros	CR
*Pterygoneurum crossidioides* W.Frey, Herrnst. & Kürschner	CR
*Pterygoneurum lamellatum* (Lindb.) Jur.	EN
*Pterygoneurum ovatum* (Hedw.) Dixon	VU
*Pterygoneurum squamosum* Segarra & Kürschner	CR
*Pterygoneurum subsessile* (Brid.) Jur.	EN
*Ptycostomum capillare* (Hedw.) *Holyoak & N.Pedersen*	LC
*Ptychostomum creberrimum* (Taylor) J.R.Spence & H.P.Ramsay	LC
*Ptychostomum imbricatulum (Müll.Hal.) Holyoak & N.Pedersen*	LC
*Syntrichia calcicola* J.J.Amann	NT
*Syntrichia ruraliformis* (Besch.) Cardot	VU
*Syntrichia ruralis* (Hedw.) F.Weber & D.Mohr	LC
*Tortella squarrosa* (Brid.) Limpr.	VU
*Tortula acaulon* (With.) R.H.Zander var. *acaulon*	LC
*Tortula acaulon* var. *pilifera* (Lindb.) R.H.Zander	NT
*Tortula brevissima* Schiffn.	*CR
*Tortula caucasica* Broth.	VU
*Tortula lindbergii* Broth.	VU
*Tortula muralis* Hedw. subsp. *muralis* var. *muralis*	LC
*Tortula protobryoides* R.H.Zander	VU
*Tortula truncata* (Hedw.) Mitt.	LC
*Weissia condensa* (Voit) Lindb.	VU
*Weissia controversa* Hedw.	LC
*Weissia longifolia* Mitt.	VU

**Table A2:** Synoptic table of syntaxa observed at the Pannonian loess cliffs of Austria; cluster 1: *Eurhynchietum schleicheri* Waldh. 1944 *didymodontetosum cordati* subass. nov., cluster 2: *Didymodontetum glauci* Ahrens ex Marst. 2015 *didymodontetosum cordati* subass nov., cluster 3: *Didymodonto-Acaulonetum triquetri* ass.nov., cluster 4: *Aloinetum rigidae* Stod. 1937, cluster 5: *Aloinetum rigidae-Aloinetosum obliquifoliae* subass nov., cluster 6: *Hilpertio velenovskyi–Pterygoneuretum compacti* Kürschner & Pócs 2002; constancy in %; nomenclature follows Hodgett et al. (2020; [55]); species with only one occurrence are not listed here; they can be seen in the tables in the Supplementary Materials.

Species/Cluster	1	2	3	4	5	6
	constancy					
***Character and differential species***						
*Oxyrrhynchium schleicheri*	83	0	0	0	0	0
*Oxyrrhynchium hians*	67	0	8.3	0	0	0
*Brachythecium rutabulum*	67	0	4.2	0	0	0
*Amblystegium serpens*	67	0	0	0	0	0
*Didymodon glaucus*	0	100	0	0	0	0
*Acaulon triquetrum*	0	25	54	3.8	18	14
*Bryum dichotomum*	0	0	46	3.8	9.1	0
*Didymodon acutus*	0	0	33	0	0	0
*Tortula brevissima*	0	0	25	3.8	0	7.1
*Homalothecium lutescens*	0	0	25	0	0	0
*Aloina rigida*	17	25	33	88	100	14
*Aloina obliquifolia*	0	0	4.2	0	100	21
*Hilpertia velenovskyi*	0	0	0	0	0	50
*Pterygoneurum compactum*	0	0	0	0	0	50
*Didymodon vinealis*	0	0	8.3	0	0	29
*Pterygoneurm squamosum*	0	0	0	0	0	14
*Didymodon cordatus*	83	100	96	100	100	93
***Grimmaldion***						
*Tortula lindbergii*	50	75	79	46	64	43
*Aloina ambigua*	50	75	79	12	18	71
*Streblotrichum convolutum var. convolutum*	50	50	79	23	18	7.1
*Pterygoneurum lamellatum*		25	54	27	45	57
*Pterygoneurum subsessile*	0	50	29	23	27	21
*Tortula acaulon var. pilifera*	0	50	29	7.7	18	29
*Pseudocrossidium hornschuchianum*	0	75	29	3.8	9.1	7.1
*Aloina brevirostris*	0	0	13	12	9.1	21
*Pterygoneurum crossidioides*	0	0	17	3.8	0	14
*Tortula protobryoides*	0	0	8.3	3.8	0	0
***Barbuletalia***						
*Pterygoneurum ovatum*	50	50	88	65	73	93
*Barbula unguiculata*	83	50	67	12	36	7.1
*Abietinella abietina var. abietina*	33	25	17	0	9.1	0
*Ptychostomum imbricatulum*	17	25	46	15	0	29
*Tortula acaulon var. acaulon*	33	0	42	0	9.1	0
*Syntrichia ruralis*	17	25	17	0	0	0
*Ptychostomum capillare*	17	0	13	3.8	0	0
*Didymodon fallax*	50	0	21	0	0	0
***Others***						
*Bryum argenteum*	50	75	92	35	73	64
*Didymodon rigidulus*	50	25	46	35	36	21
*Tortula muralis subsp. muralis var. muralis*	17	50	38	15	36	7.1
*Bryum violaceum*	0	25	8.3	0	0	7.1
*Bryum radiculosum*	0	25	8.3	0	0	7.1
*Grimmia pulvinata*	0	25	13	0	0	0
*Tortula caucasica*	0	25	13	0	0	0
*Dicranella howei*	0	25	4.2	0	0	0
*Funaria hygrometrica*	17	0	4.2	0	0	0
*Syntrichia ruraliformis*	0	0	4.2	3.8	0	0
*Entodon concinnus*	0	0	4.2	0	9.1	0
*Campyliadelphus chrysophyllus*	17	25	0	0	0	0
