# Supplementary material for: Bryophytes of the Loess Cliffs in the Pannonian Area of Austria
Source: Plants (Basel). 2025 Oct 10;14(20):3128. doi: 10.3390/plants14203128 (PMC12566642; doi:10.3390/plants14203128)
Supplement: Supplementary file 1 [file plants-14-03128-s001.zip › Table S1.pdf]

Table S1. *Eurhynchietum schleicheri* Waldh. 1944 *didymodontetosum cordati* subass. nov. (cluster 1), *Didymodontetum glauci* Ahrens ex Marst. 2015 *didymodontetosum cordati* subass. nov. (cluster 2), fragment community (cluster 0); C%–constancy in % of a species in the associated community, relevé number in bold: nomenclatoral type.

**cluster**	0	1	1	1	1	1	1	C%	2	2	2	2	C%
**Relevé number**	82	53	41	**23**	86	69	37	S	78	2	**9**	66	S
Character and differential species													
Oxyrrhynchium schleicheri	.	.	10	10	2	10	2	83					
Oxyrrhynchium hians	.	.	50	10	.	10	50	67					
Brachythecium rutabulum	.	.	10	10	10	.	10	67					
Amblystegium serpens	.	50	10	10	10	.	.	67					
Didymodon glaucus	.								2	10	10	2	100
Didymodon cordatus	.	.	50	50	10	50	10	83	50	50	50	2	100
Pseudocrossidium hornschuchianum	.	.	.	.	.	.	.		2	.	2	2	75
Grimmaldion													
Tortula lindbergii	.	.	.	10	10	.	10	50	2	.	10	10	75
Aloina ambigua	.	2	.	10	.	2	.	50	2	.	2	10	75
Streblotrichum convolutum var. *convolutum*	.	50	.	10	.	.	10	50	2	.	10	.	50
Pterygoneurum subsessile	.	.	.	.	.	.	.		2	.	.	2	50
Barbuletalia													
Bryum argenteum	.	.	.	10	10	.	10	50	2	.	10	50	75
Barbula unguiculata	.	50	10	50	10	.	50	83	2	.	2	.	50
Pterygoneurum ovatum	.	.	.	.	10	10	10	50	2	.	.	10	50
Tortula muralis subsp. *muralis* var. *muralis*	.	.	10	.	.	.	.	17	2	.	.	50	50
Didymodon rigidulus	.	.	10	10	.	2	.	50	2	.	.	.	25
Abietinella abietina var. *abietina*	.	10	.	.	.	10	.	33	.	.	10	.	25
Tortula acaulon var. *acaulon*	.	.	.	.	2	.	10	33					
Didymodon fallax	10	10	.	.	.	10	10	50					
Aloina rigida	.	.	.	.	10	.	.	17	.	.	50	.	25
Pterygoneurum lamellatum	.	.	.	.	.	.	.	.	.	.	.	2	25
Acaulon triquetrum	.	.	.	.	.	.	.	.	.	.	.	2	25
Others													
Ptychostomum imbricatulum	10	.	.	10	.	.	.	17	.	.	2	.	25
Tortula truncata	.	.	.	10	.	.	.	17	.	.	.		.
Ptychostomum capillare	.	.	.	10	.	.	.	17	.	.	.	.	.
Funaria hygrometrica	.	.	.	.	.	10	.	17	.	.	.	.	.
Syntrichia ruralis	.	.	.	.	10	.	.	17	.	.	.	10	25
Tortula caucasica	10	.	.	.	.	.	.	.	.	.	.	10	25
Bryum violaceum	.	.	.	.	.	.	.	.	.	.	.	2	25
Grimmia pulvinata	.	.	.	.	.	.	.	.	.	.	.	2	25
Bryum radiculosum	.	.	.	.	.	.	.	.	.	.	.	2	25
Dicranella howei	.	.	.	.	.	.	.	.	.	.	.	2	25
Didymodon ferrugineus	.	.	.	.	.	10	.	17	.	.	.	.	.

Campyliadelphus chrysophyllus	.	.	.	10	.	.	.	17	.	.	.	.	25
Plagiomnium undulatum	.	.	.	.	.	.	10	17	.	.	.	.	.
Brachytheciastrum velutinum	.	.	50	.	.	.	.	17	.	.	.	.	.
