# Supplementary material for: Bryophytes of the Loess Cliffs in the Pannonian Area of Austria
Source: Plants (Basel). 2025 Oct 10;14(20):3128. doi: 10.3390/plants14203128 (PMC12566642; doi:10.3390/plants14203128)
Supplement: Supplementary file 1 [file plants-14-03128-s001.zip › Table S2.pdf]

Table S2. *Didymodonto-Acaulonetum triquetri* ass. nov, C%–constancy in % of a species in the community, relevé number in bold: nomenclatoral type.

Relevé number	51	83	30	61	2	80	68	35	74	56	85	36	**43**	84	57	38	7	49	11	19	1	61	41	31	C%
cluster	3	3	3	3	3	3	3	3	3	3	3	3	3	3	3	3	3	3	3	3	3	3	3	3	
Character species																									
Didymodon cordatus	2	2	3	2	3	3	2	3	3	3	2	3	3	2	.	3	2	3	2	2	2	3	3	3	96
Acaulon triquetrum	.	1	1	1	.	.	.	.	2	.	.	2	2	1	2	2	2	1	2	.	.	.	2	.	54
Bryum dichotomum	.	.	.	.	.	.	.	2	2	.	.	1	1	.	2	2	2	2	2	.	2	.	2	.	46
Didymodon acutus	2	2	1	.	.	.	.	.	.	.	2	.	.	2	2	1	.	.	2	.	.	.	.	.	33
Tortula brevissima	.	.	.	.	.	.	.	.	1	.	1	.	1	.	.	.	.	1	1	.	.	.	.	1	25
Differentiating species																									
Homalothecium lutescens	2	.	.	2	.	3	.	3	2	2	.	.	.	.	.	.	.	.	.	.	.	.	.	.	25
Grimmaldion																									
Tortula lindbergii	.	.	2	.	2	2	2	2	3	.	1	2	2	.	2	2	2	2	2	2	2	2	2	3	79
Aloina ambigua	1	1	2	.	2	3	2	2	2	3	2	2	.	3	2	2	2	2	2	.	.	2	2	.	79
Streblotrichum convolutum var. convolutum	.	2	2	.	2	2	3	3	3	2	2	2	3	3	2	2	2	2	2	.	.	.	3	2	79
Pterygoneurum lamellatum	.	1	1	.	.	1	.	1	2	1	1	1	2	2	.	2	.	.	2	.	1	.	.	.	54
Aloina rigida	2	2	2	.	.	.	.	.	.	.	2	2	.	1	.	.	.	1	2	.	.	.	.	.	33
Pseudocrossidium hornschuchianum	.	1	.	.	.	.	1	.	.	.	.	1	1	1	.	.	.	1	1	.	.	.	.	.	29
Pterygoneurum subsessile	.	.	1	.	.	.	.	.	1	.	.	1	.	.	.	.	1	.	1	.	1	.	1	.	29
Tortula acaulon var. pilifera	.	2	1	.	.	.	.	.	.	.	.	.	.	.	2	.	2	1	.	.	2	.	.	2	29
Microbryum curvicollum	.	1	.	.	1	.	.	.	.	.	.	.	1	.	.	.	.	.	.	.	.	.	1	.	17
Pterygoneurum crossidioides	.	.	.	.	.	.	.	.	.	1	.	.	1	.	.	.	.	.	1	.	.	.	1	.	17
Microbryum floerkeanum	.	.	1	.	.	.	.	.	.	.	1	.	.	.	.	.	.	.	1	.	.	.	.	.	13
Aloina brevirostris	.	.	.	.	.	.	.	.	.	2	.	.	1	.	.	.	.	.	1	.	.	.	.	.	13
Didymodon vinealis	.	1	.	.	.	.	.	.	.	.	.	.	.	1	.	.	.	.	.	.	.	.	.	.	8.3
Didymodon insulanus	.	.	.	.	.	.	.	.	.	.	.	.	.	.	.	.	.	.	1	.	.	.	.	2	8.3

Weissia condensa	.	.	.	.	2	.	.	.	.	.	.	.	.	.	.	.	2	.	.	.	.	.	.	.	8.3
Weissia longifolia	.	2	.	.	.	.	.	.	.	.	.	.	.	.	.	.	.	.	2	.	.	.	.	.	8.3
Tortula protobryoides	.	2	.	.	.	.	.	.	.	.	.	.	.	.	.	.	.	.	1	.	.	.	.	.	8.3
Aloina obliquifolia	.	1	.	.	.	.	.	.	.	.	.	.	.	.	.	.	.		.	.	.	.	.	.	4.2
Pseudocrossidium revolutum	.	.	.	.	.	.	.	.	.	.	.	1	.	.	.	.	.	.	.	.	.	.	.	.	4.2
Barbuletalia																									
Pterygoneurum ovatum	2	2	2	2	2	.	.	3	3	1	2	2	2	2	.	3	2	2	2	2	2	2	2	3	88
Barbula unguiculata	2	.	.	.	2	3	3	2	3	1	.	3	2	3	.	2	2	2	2	.	.	3	.	2	67
Tortula acaulon var. acaulon	.	2	1	.	2	.	.	.	.	.	.	.	.	.	2	2	2	1	2	2	.	.	.	3	42
Ptychostomum imbricatulum	.	.	.	.	2	.	.	2	2	.	.	2	.	2	.	2	2	.	2	2	2	.	.	3	46
Bryum radiculosum	.	.	.	.	.	.	.	1	.	.	.	.	.	.	.	.	.	.	1	.	.	.	.	.	8.3
Ptychostomum creberrimum	.	.	.	2	2	.	.	.	.	.	.	.	.	.	.	.	.	.	.	.	.	.	.	.	8.3
Microbryum starckeanum	.	.	.	.	.	.	.	.	.	.	.	.	.	2	.	.	.	.	.	.	.	.	.	.	4.2
Others																									
Bryum argenteum	.	2	2	2	2	2	.	2	3	2	2	3	2	2	2	2	2	2	2	2	2	2	2	3	92
Didymodon rigidulus	.	.	1	.	.	.	.	1	2	2	2	.	.	.	.	1	.	2	2	.	1	.	1	2	46
Tortula muralis subsp. muralis var. muralis	.	.	.	2	.	.	2	.	.	2	2	1	.	3	.	.	.	.	2	2	.	.	.	2	38
Didymodon fallax	.	.	.	.	2	.	.	2	2	2	.	.	.	.	.	.	.	.	.	.	1	.	.	.	21
Abietinella abietina var. abietina	.	2	.	.	.	2	.	.	.	1	.	.	.	.	.	.	.	.	2	.	.	.	.	.	17
Syntrichia ruralis	2	2	.	.	.	.	.	.	.	1	2	.	.	.	.	.	.	.	.	.	.	.	.	.	17
Ptychostomum capillare	.	.	.	.	.	2	.	.	.	.	.	.	.	.	.	.	.	.	2	.	.	.	.	2	13
Tortula caucasica	2	2	.	.	.	.	.	.	.	.	.	1	.	.	.	.	.	.	.	.	.	.	.	.	13
Grimmia pulvinata	.	.	.	2	.	.	.	.	.	.	.	1	.	1	.	.	.	.	.	.	.	.	.	.	13
Orthotrichum anomalum	.	.	.	2	.	.	.	.	.	.	.	.	.	.	.	.	.	.	1	.	.	.	.	.	8.3
Oxyrrhynchium hians	.	.	.	.	2	3	.	.	.	.	.	.	.	.	.	.	.	.	.	.	.	.	.	.	8.3
Bryum violaceum	.	.	.	.	.	.	.	1	.	.	.	.	.	.	.	.	.	.	2	.	.	.	.	.	8.3
Acaulon muticum	.	.	.	.	.	.	.	.	.	.	.	.	.	.	2	.	.	.	.	.	2	.	.	.	8.3
Encalypta vulgaris	.	.	.	.	.	.	.	.	.	.	.	.	.	.	.	.	.	.	1	.	.	.	.	.	4.2
Dicranella howei	.	.	.	.	1	.	.	.	.	.	.	.	.	.	.	.	.	.	.	.	.	.	.	.	4.2

Encalypta streptocarpa	.	.	.	.	.	50	.	.	.	.	.	.	.	.	.	.	.	.	.	.	.	.	.	.	4.2
Pottiopsis caespitosa	.	.	.	.	.	.	.	.	.	.	.	.	.	.	.	.	.	.	2	.	.	.	.	.	4.2
Plagiomnium cuspidatum	.	.	.	.	.	10	.	.	.	.	.	.	.	.	.	.	.	.	.	.	.	.	.	.	4.2
Brachythecium glareosum	10	.	.	.	.	.	.	.	.	.	.	.	.	.	.	.	.	.	.	.	.	.	.	.	4.2
Brachythecium albicans	.	.	.	.	10	.	.	.	.	.	.	.	.	.	.	.	.	.	.	.	.	.	.	.	4.2
Weissia controversa	10	.	.	.	.	.	.	.	.	.	.	.	.	.	.	.	.	.	.	.	.	.	.	.	4.2
Ceratodon purpureus	.	.	.	.	10	.	.	.	.	.	.	.	.	.	.	.	.	.	.	.	.	.	.	.	4.2
Tortella squarrosa	.	10	.	.	.	.	.	.	.	.	.	.	.	.	.	.	.	.	.	.	.	.	.	.	4.2
Brachythecium rutabulum	.	.	.	.	.	.	.	.	2	.	.	.	.	.	.	.	.	.	.	.	.	.	.	.	4.2
Funaria hygrometrica	.	.	2	.	.	.	.	.	.	.	.	.	.	.	.	.	.	.	.	.	.	.	.	.	4.2
Bryum klinggraeffii	.	10	.	.	.	.	.	.	.	.	.	.	.	.	.	.	.	.	.	.	.	.	.	.	4.2
Syntrichia calcicola	.	.	.	.	.	.	.	.	.	2	.	.	.	.	.	.	.	.	.	.	.	.	.	.	4.2
Brachythecium campestre	.	.	.	.	.	.	.	.	.	.	.	.	.	.	10	.	.	.	.	.	.	.	.	.	4.2
Entodon concinnus	.	10	.	.	.	.	.	.	.	.	.	.	.	.	.	.	.	.	.	.	.	.	.	.	4.2
Syntrichia ruraliformis	.	.	.	.	.	.	.	.	.	.	.	.	.	.	.	.	.	.	2	.	.	.	.	.	4.2
Ditrichum pusillum	.	.	.	.	.	.	.	.	.	.	.	.	.	.	.	.	.	.	.	.	2	.	.	.	4.2
