# Supplementary material for: Bryophytes of the Loess Cliffs in the Pannonian Area of Austria
Source: Plants (Basel). 2025 Oct 10;14(20):3128. doi: 10.3390/plants14203128 (PMC12566642; doi:10.3390/plants14203128)
Supplement: Supplementary file 1 [file plants-14-03128-s001.zip › Table S3.pdf]

Table S3. *Aloinetum rigidae* Stod. 1937 (cluster 4); *Aloinetum rigidae-Aloinetosum obliquifoliae* subass nov. (cluster 5); C%–constancy in % of a species in the associated community, relevé number in bold: nomenclatoral type.

Relevé number	81	18	67	44	17	16	77	16	79	19	2	34	31	6	46	45	33	54	47	3	15	71	71	60	17	63	C%	75	18	65	**14**	58	5	4	40	48	39	15	C%
Cluster	4	4	4	4	4	4	4	4	4	4	4	4	4	4	4	4	4	4	4	4	4	4	4	4	4	4		5	5	5	5	5	5	5	5	5	5	5	
Character species																																							
Aloina rigida	.	1	2	2	1	2	2	2	2	1	2	2	1	1	2	.	1	1	1	.	2	1	2	3	3	2	88	3	3	3	1	2	2	2	2	2	1	1	100
Didymodon cordatus	1	2	3	3	3	3	3	3	3	3	3	3	3	3	3	3	3	2	3	3	3	3	2	3	3	2	100	3	3	3	3	3	3	3	3	2	3	3	100
Tortula lindbergii	.	.	.	.	1	.	2	2	2	2	2	2	2	.	2	.	1	.	.	1	.	2	.	.	.	.	46	2	2	.	2	2	.	1	2	2	.	.	64
Aloina obliquifolia	.	.	.	.	.	.	.	.	.	.	.	.	.	.	.	.	.	.	.	.	.	.	.	.	.	.		1	2	1	2	2	2	1	2	1	1	1	100
Grimmaldion																																							
Pterygoneurum lamellatum	.	.	.	.	.	.	.	.	.	1	.	.	2	.	.	1	.	.	.	2	.	2	2	.	.	3	27	2	.	1	1	.	2	.	.	.	.	2	45
Pterygoneurum subsessile	1	.	.	.	.	2	.	.	.	.	.	.	.	.	.	.	1	.	.	.	.	1	.	.	1	1	23	.	.	.	1	.	.	.	1	1	.	.	27
Streblotrichum convolutum var. convolutum	.	2	3	1	3	.	.	.	2	1	.	.	.	.	.	.	.	.	.	.	.	.	.	.	.	.	23	.	.	.	1	.	.	.	.	2	.	.	18
Aloina ambigua	.	.		.	.	.	.	.	.		.		.	.	.	.		.	.	.	.	.	2	1	2		12	.	.	.	.	1	.	.	1	.		.	18
Acaulon triquetrum	.	.	.	.	.	.	.	.	.	.	.	.	.	.	.	.	.	.	.	.	.	.	.	.	.	1	4	.	.	.	1	1	.	.	.	.	.	.	18
Tortula acaulon var. pilifera	.	.	.	.	.	.	.	.	2	.	.	.	.	.	.	.	.	.	.	.	.	.	.	.	.	1	8	.	.	3	.	2	.	.	.	.	.	.	18
Aloina brevirostris	.	.	.	.	1	.	.	.	.	.	.	.	1	.	.	.	.	.	.	.	.	.	.	.	.	1	12	.	.	.	1	.	.	.	.	.	.	.	9
Barbulatalia																																							
Pterygoneurum ovatum	.	2	2	2	2	2	.	3	2	1	2	2	2	.	2	2	2	.	.	.	.	2	.	.	2	2	65	2	.	3	2	1	2	2	2	2	.	.	73
Barbula unguiculata	.	.	.	.	.	.	.	.	.	.	.	.	.	1	.	.	.	.	.	.	.	1	2	.	.	.	12	3	.	.	.	.	2	2	2	.	.	.	36
Ptychostomum imbricatulum	.	.	2	.	.	.	.	.	.	.	.	.	.	.	.	.	.	.	.	2	.	2	2	.	.	.	15	.	.	.	.	.	.	.	.	.	.	.	
Others																																							
Bryum argenteum	.	.	3	.	.	.	.	.	2	1	2	2	.	.	.	2	.	.	.	2	.	2	.	.	2	.	35	3	2	.	2	1	2	2	2	.	.	1	73
Didymodon rigidulus	.	2	.	1	.	.	.	2	.	1	.	.	.	.	2	1	.	.	2	2	.	2	.	.	.	.	35	.	.	.	.	.	.	1	2	2	.	1	36
Tortula muralis subsp. muralis var. muralis	.	2	2	.	.	.	.	1	.	.	.	.	.	.	.	.	.	.	1	.	.	.	.	.	.	.	15	1	2	1	.	2	.	.	.	.	.	.	36
Bryum dichotomum	1	.	.	.	.	.	.	.	.	.	.	.	.	.	.	.	.	.	.	.	.	.	.	.	.	.	4	.	.	.	.	2	.	.	.	.	.	.	10
Pseudocrossidium hornschuchianum	.	.	.	.	.	.	.	.	.	.	.	.	.	.	.	.	.	.	.	.	.	.	1	.	.	.	4	.	.	.	.	.	.	1	.	.	.	.	10
Ptychostomum capillare	.	.	.	.	.	.	.	2	.	.	.	.	.	.	.	.	.	.	.	.	.	.	.	.	.	.	4	.	.	.	.	.	.	.	.	.	.	.	
Tortula brevissima	.	1	.	.	.	.	.	.	.	.	.	.	.	.	.	.	.	.	.	.	.	.	.	.	.	.	4	.	.	.	.	.	.	.	.	.	.	.	
Tortula protobryoides	.	.	.	.	.	.	.	.	.	.	.	.	.	.	.	.	.	.	.	1	.	.	.	.	.	.	4	.	.	.	.	.	.	.	.	.	.	.	
Pterygoneurum crossidioides	.	.	.	.	.	.	1	.	.	.	.	.	.	.	.	.	.	.	.	.	.	.	.	.	.	.	4	.	.	.	.	.	.	.	.	.	.	.	
Syntrichia ruraliformis	1	.	.	.	.	.	.	.	.	.	.	.	.	.	.	.	.	.	.	.	.	.	.	.	.	.	4	.	.	.	.	.	.	.	.	.	.	.	
Entodon concinnus	.	.	.	.	.	.	.	.	.	.	.	.	.	.	.	.	.	.	.	.	.	.	.	.	.	.		.	.	.	.	.	.	.	2	.	.	.	10
Grimmia montana	.	.	.	.	.	.	.	.	.	.	.	.	.	.	.	.	.	.	.	.	.	.	.	.	.	.		.	.	2	.	.	.	.	.	.	.	.	10
Tortula acaulon var. acaulon	.	.	.	.	.	.	.	.	.	.	.	.	.	.	.	.	.	.	.	.	.	.	.	.	.	.		2	.	.	.	.	.	.	.	.	.	.	10

Abietinella abietina var. abietina	.	.	.	.	.	.	.	.	.	.	.	.	.	.	.	.	.	.	.	.	.	.	.	.	.	.	4	.	.	.	.	.	.	.	2	.	.	.	10
