# Supplementary material for: Bryophytes of the Loess Cliffs in the Pannonian Area of Austria
Source: Plants (Basel). 2025 Oct 10;14(20):3128. doi: 10.3390/plants14203128 (PMC12566642; doi:10.3390/plants14203128)
Supplement: Supplementary file 1 [file plants-14-03128-s001.zip › Table S4.pdf]

Table S4. *Hilpertio velenovskyi–Pterygoneuretum compacti* Kürschner & Pócs 2002 (cluster 6); C%–constancy in % of a species in the associated community.

Relevé number	11	70	11	76	59	14	73	8	13	55	11	3	51	64	**C%**
Cluster	6	6	6	6	6	6	6	6	6	6	6	6	6	6	
Character species															
Hilpertia velenovskyi	.	.	1	1	.	.	1	1	2	.	.	.	1	1	50
Pterygoneurum compactum	2	1	.	.	.	1	1	1	.	1	1	.	.	.	50
Didymodon vinealis	.	2	.	.	.	.	.	.	.	.	.	2	1	2	29
Pterygoneurum squamosum	.	.	.	.	1	.	.	.	.	.	.	2	.	.	14
Grimmaldion															
Didymodon cordatus	3	2	3	3	2	3	3	3	3	3	3	3	.	2	93
Aloina ambigua	.	2	2	2	.	2	2	3	.	2	.	3	2	3	71
Pterygoneurum lamellatum	.	3	1	1	.	.	1	.	2	2	.	2	.	2	57
Tortula lindbergii	.	1	2	.	2	.	2	.	.	2	.	.	1	.	43
Tortula acaulon var. pilifera	.	.	1	1	2	.	.	.	.	.	.	.	1	.	29
Aloina brevirostris	.	.	.	.	.	1	.	.	.	.	.	.	1	1	21
Aloina obliquifolia	.	.	1	.	.	.	1	.	.	.	.	1	.	.	21
Pterygoneurum subsessile	.	1	.	.	1	.	.	.	.	1	.	.	.	.	21
Pterygoneurum crossidioides	.	1	.	1	.	.	.	.	.	.	.	.	.	.	14
Aloina rigida	.	.	2	1	.	.	.	.	.	.	.	.	.	.	14
Acaulon triquetrum	.	.	.	1	1	.	.	.	.	.	.	.	.	.	14
Barbuletalia															
Pterygoneurum ovatum	2	2	2	2	1	2	2	2	.	2	2	2	1	2	93
Bryum argenteum	2	.	2	1	.	2	.	2	2	.	2	3	.	2	64
Ptychostomum imbricatulum	.	2	.	.	.	.	1	.	.	.	.	2	2	.	29
Others															
Didymodon rigidulus	.	.	.	.	.	.	.	.	.	2	.	2	.	2	21
Barbula unguiculata	.	.	.	.	.	.	.	.	.	.	.	.	.	2	7
Aloina aloides	.	1	.	.	.	.	.	.	.	.	.	.	.	.	7
Streblotrichum convolutum var. convolutum	.	.	.	.	.	.	.	.	.	.	.	.	.	2	7
Pseudocrossidium hornschuchianum	.	.	.	.	.	.	.	.	.	.	.	.	1	.	7
Tortula brevissima	.	.	.	.	.	.	.	.	.	.	.	.	.	1	7
Bryum violaceum	.	.	.	.	.	.	1	.	.	.	.	.	.	.	7
Bryum radiculosum	.	.	.	.	.	.	1	.	.	.	.	.	.	.	7
Tortula muralis subsp. muralis var. muralis	.	.	.	.	3	.	.	.	.	.	.	.	.	.	7
